# Tetra­kis[3,5-bis­(trifluoro­meth­yl)phenyl]tin(IV)

**DOI:** 10.1107/S1600536809019588

**Published:** 2009-06-06

**Authors:** Daniel Foucher, Damion Miles, Alan J. Lough

**Affiliations:** a Department of Chemistry and Biology, Ryerson University, Toronto, Ontario, Canada M5B 2K3; b Department of Chemistry, University of Toronto, Toronto, Ontario, Canada M5S 3H6

## Abstract

The title mol­ecule, [Sn(C_8_H_3_F_6_)_4_], lies on a twofold rotation axis with the Sn^IV^ ion in a distorted tetra­hedral coordination environment. Both –CF_3_ groups attached to one of the unique benzene rings are disordered over two sets of sites, with the ratios of refined occupancies being 0.719 (14):0.281 (14) and 0.63 (5):0.37 (5).

## Related literature

For synthesis of the title compound, see King *et al.* (1986 ▶). Additional preparative details of similar compounds are given by Lu & Tilley (2000 ▶). For related crystal structures, see: Young *et al.* (2005 ▶); Smith *et al.* (1994 ▶); Wharf & Simard (1997 ▶). For further details of geometric distortions in related compounds, see Charissé *et al.* (1998 ▶).
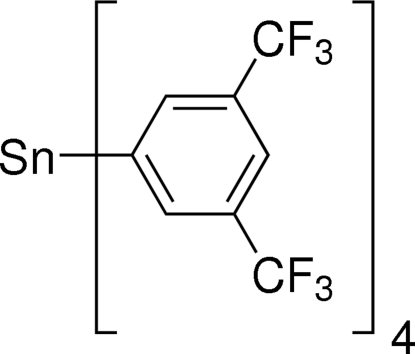


## Experimental

### 

#### Crystal data

[Sn(C_8_H_3_F_6_)_4_]
*M* *_r_* = 971.11
Monoclinic, 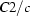

*a* = 17.3506 (8) Å
*b* = 20.8038 (11) Å
*c* = 9.8944 (3) Å
β = 109.998 (3)°
*V* = 3356.1 (3) Å^3^
*Z* = 4
Mo *K*α radiation
μ = 0.92 mm^−1^
*T* = 150 K
0.28 × 0.24 × 0.12 mm

#### Data collection

Nonius KappaCCD diffractometer
Absorption correction: multi-scan (*SORTAV*; Blessing, 1995 ▶) *T*_min_ = 0.798, *T*_max_ = 0.897
10930 measured reflections
3818 independent reflections
3142 reflections with *I* > 2σ(*I*)
*R*_int_ = 0.038

#### Refinement

*R*[*F* ^2^ > 2σ(*F* ^2^)] = 0.041
*wR*(*F* ^2^) = 0.097
*S* = 1.06
3818 reflections
314 parameters
211 restraints
H-atom parameters constrained
Δρ_max_ = 1.80 e Å^−3^
Δρ_min_ = −0.69 e Å^−3^

### 

Data collection: *COLLECT* (Nonius, 2002 ▶); cell refinement: *DENZO-SMN* (Otwinowski & Minor, 1997 ▶); data reduction: *DENZO-SMN*; program(s) used to solve structure: *SIR92* (Altomare *et al.*, 1994 ▶); program(s) used to refine structure: *SHELXTL* (Sheldrick, 2008 ▶); molecular graphics: *PLATON* (Spek, 2009 ▶); software used to prepare material for publication: *SHELXTL*.

## Supplementary Material

Crystal structure: contains datablocks global, I. DOI: 10.1107/S1600536809019588/pk2165sup1.cif

Structure factors: contains datablocks I. DOI: 10.1107/S1600536809019588/pk2165Isup2.hkl

Additional supplementary materials:  crystallographic information; 3D view; checkCIF report

## Figures and Tables

**Table:** 

Sn1—C9	2.146 (3)
Sn1—C1	2.150 (3)

**Table:** 

C9—Sn1—C9^i^	109.73 (16)
C9—Sn1—C1	104.69 (11)
C9—Sn1—C1^i^	108.35 (11)
C1—Sn1—C1^i^	120.82 (17)

Symmetry code: (i) 
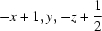
.

**Fig. 1.**
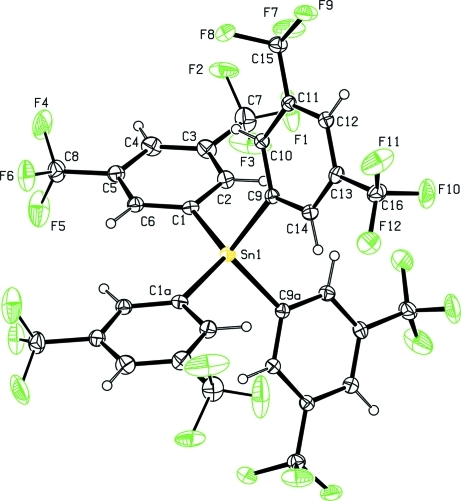
The molecular structure of the title compound with displacement ellipsoids drawn at the 30% probabilty level. The minor comonent of disorder is not shown [symmetry code (a): -x + 1, y, -z + 1/2].

## supplementary crystallographic information

### Comment

The preparation of polymerizable dialkyl or diaryl tin monomers bearing either chlorine or hydride groups (Lu & Tilley, 2000) is accessed through the initial comportionation reactions involving the tetraalkyl- or tetraryltin(IV) compounds and tin(IV) tetrachloride. The incorporation of perfluorinated species in the backbone of polystannanes should by design impart an improved stability towards nucleophilic attack. Our interest in the distortions from tetrahedral geometry of other tin aryl compounds (Charissé *et al.*, 1998), prompted us to determine the crystal structure of the title compound which was previously synthesized by King *et al.* (1986).

The title molecule (Fig. 1) lies on a twofold rotation axis. The Sn^IV^ ion is in a distorted tetrahedral coordination environment (Table 1). The angular disortion from the ideal values of 109.5° is most likely a consequence of the steric crowding caused by the 3,5 substitution of the bulky trifluoromethyl groups on the benzene rings. The Sn—C bond distances in the title compound are the same within experimental error and are comparable to those in the *para*-substituted and *meta*-substituted tetrakis[(trifluoromethyl)phenyl]stannane structures (Young *et al.*, 2005; Smith *et al.*, 1994) but are significantly longer than the Sn—C bonds in the related triaryltin(IV)chloride compounds (Wharf & Simard, 1997).

### Experimental

The title compound was prepared from the refluxing Grignard reaction of 3,5-trifluoromethylphenyl magnesium bromide (12.5 mmol) in ether with anhydrous tin tetrachloride (3.125 mmol). The reaction mixture was refluxed overnight, cooled and filtered to remove salts. The crude compound was purified first by sublimation, and then recrystallization from ether to yield long large needles suitable for X-ray diffraction. Yield 1.33 g, 44%. m.p. 426 K (literature 436 K; King *et al.*, 1986).

### Refinement

H atoms were placed in calculated positions with C—H = 0.95 Å and included in a riding-motion approximation with *U*_iso_(H) = 1.2*U*_eq_(C). Both –CF_3_ groups attached to one of the unique benzene rings are disordered over two sets of sites with the ratios of refined occupancies being 0.719 (14):0.281 (14) for F1/F2/F3:F1A/F2A/F3A, and 0.63 (5):0.37 (5) for F4/F5/F6:F4A/F5A/F6A. The SADI and SIMU commands in *SHELXL* (Sheldrick, 2008) were used to restrain the disorder model.

### Crystal data

**Table:**

[Sn(C_8_H_3_F_6_)_4_]	*F*(000) = 1880
*M**_r_* = 971.11	*D*_x_ = 1.922 Mg m^−^^3^
Monoclinic, *C*2/*c*	Mo *K*α radiation, λ = 0.71073 Å
Hall symbol: -C 2yc	Cell parameters from 10930 reflections
*a* = 17.3506 (8) Å	θ = 2.9–27.5°
*b* = 20.8038 (11) Å	µ = 0.92 mm^−^^1^
*c* = 9.8944 (3) Å	*T* = 150 K
β = 109.998 (3)°	Block, colourless
*V* = 3356.1 (3) Å^3^	0.28 × 0.24 × 0.12 mm
*Z* = 4	

### Data collection

**Table:**

Nonius KappaCCD diffractometer	3818 independent reflections
Radiation source: fine-focus sealed tube	3142 reflections with *I* > 2σ(*I*)
graphite	*R*_int_ = 0.038
Detector resolution: 9 pixels mm^-1^	θ_max_ = 27.5°, θ_min_ = 2.9°
φ scans and ω scans with κ offsets	*h* = −22→20
Absorption correction: multi-scan (*SORTAV*; Blessing, 1995)	*k* = −24→26
*T*_min_ = 0.798, *T*_max_ = 0.897	*l* = −10→12
10930 measured reflections	

### Refinement

**Table:**

Refinement on *F*^2^	Primary atom site location: structure-invariant direct methods
Least-squares matrix: full	Secondary atom site location: difference Fourier map
*R*[*F*^2^ > 2σ(*F*^2^)] = 0.041	Hydrogen site location: inferred from neighbouring sites
*wR*(*F*^2^) = 0.097	H-atom parameters constrained
*S* = 1.06	*w* = 1/[σ^2^(*F*_o_^2^) + (0.0382*P*)^2^ + 9.7798*P*] where *P* = (*F*_o_^2^ + 2*F*_c_^2^)/3
3818 reflections	(Δ/σ)_max_ = 0.001
314 parameters	Δρ_max_ = 1.80 e Å^−^^3^
211 restraints	Δρ_min_ = −0.69 e Å^−^^3^

### Special details

**Table:**

Geometry. All e.s.d.'s (except the e.s.d. in the dihedral angle between two l.s. planes) are estimated using the full covariance matrix. The cell e.s.d.'s are taken into account individually in the estimation of e.s.d.'s in distances, angles and torsion angles; correlations between e.s.d.'s in cell parameters are only used when they are defined by crystal symmetry. An approximate (isotropic) treatment of cell e.s.d.'s is used for estimating e.s.d.'s involving l.s. planes.
Refinement. Refinement of *F*^2^ against ALL reflections. The weighted *R*-factor *wR* and goodness of fit *S* are based on *F*^2^, conventional *R*-factors *R* are based on *F*, with *F* set to zero for negative *F*^2^. The threshold expression of *F*^2^ > σ(*F*^2^) is used only for calculating *R*-factors(gt) *etc*. and is not relevant to the choice of reflections for refinement. *R*-factors based on *F*^2^ are statistically about twice as large as those based on *F*, and *R*- factors based on ALL data will be even larger.

### Fractional atomic coordinates and isotropic or equivalent isotropic displacement parameters (Å^2^)

**Table:**

	*x*	*y*	*z*	*U*_iso_*/*U*_eq_	Occ. (<1)
Sn1	0.5000	0.204875 (15)	0.2500	0.02382 (11)	
C1	0.44875 (18)	0.25591 (16)	0.3883 (3)	0.0241 (7)	
C2	0.4192 (2)	0.21779 (17)	0.4754 (3)	0.0308 (7)	
H2A	0.4214	0.1723	0.4688	0.037*	
C3	0.3864 (2)	0.24535 (19)	0.5721 (4)	0.0358 (8)	
C4	0.3826 (2)	0.31171 (19)	0.5823 (4)	0.0376 (9)	
H4A	0.3609	0.3307	0.6489	0.045*	
C5	0.4109 (2)	0.35008 (17)	0.4942 (4)	0.0301 (7)	
C6	0.44410 (19)	0.32238 (16)	0.3985 (3)	0.0269 (7)	
H6A	0.4638	0.3491	0.3395	0.032*	
C7	0.3561 (3)	0.2031 (2)	0.6667 (5)	0.0518 (11)	
C8	0.4071 (3)	0.42168 (19)	0.5053 (4)	0.0420 (9)	
C9	0.40171 (18)	0.14550 (15)	0.1187 (3)	0.0225 (6)	
C10	0.32103 (18)	0.15735 (15)	0.1095 (3)	0.0232 (6)	
H10A	0.3099	0.1908	0.1654	0.028*	
C11	0.25646 (19)	0.12090 (15)	0.0196 (3)	0.0234 (6)	
C12	0.27116 (19)	0.07233 (15)	−0.0643 (3)	0.0254 (7)	
H12A	0.2272	0.0477	−0.1264	0.030*	
C13	0.3514 (2)	0.06033 (16)	−0.0559 (3)	0.0273 (7)	
C14	0.4159 (2)	0.09614 (16)	0.0350 (3)	0.0270 (7)	
H14A	0.4705	0.0869	0.0402	0.032*	
C15	0.1714 (2)	0.13382 (18)	0.0164 (4)	0.0315 (8)	
C16	0.3678 (2)	0.00755 (19)	−0.1447 (4)	0.0402 (9)	
F1	0.3387 (8)	0.1480 (5)	0.6254 (12)	0.110 (5)	0.510 (14)
F2	0.2865 (4)	0.2297 (5)	0.6815 (9)	0.060 (3)	0.510 (14)
F3	0.4062 (4)	0.2039 (7)	0.8017 (6)	0.098 (4)	0.510 (14)
F1A	0.3073 (6)	0.1557 (6)	0.5899 (12)	0.074 (3)	0.490 (14)
F2A	0.3197 (9)	0.2296 (5)	0.7385 (15)	0.151 (6)	0.490 (14)
F3A	0.4188 (4)	0.1697 (6)	0.7542 (13)	0.122 (5)	0.490 (14)
F4	0.3437 (13)	0.4405 (10)	0.539 (3)	0.113 (5)	0.62 (5)
F5	0.4721 (11)	0.4474 (7)	0.5986 (12)	0.078 (4)	0.62 (5)
F6	0.3982 (8)	0.4522 (6)	0.3803 (8)	0.062 (2)	0.62 (5)
F4A	0.3656 (14)	0.4402 (16)	0.587 (3)	0.081 (5)	0.38 (5)
F5A	0.4839 (9)	0.4429 (9)	0.574 (2)	0.064 (4)	0.38 (5)
F6A	0.3803 (18)	0.4473 (12)	0.3853 (13)	0.094 (8)	0.38 (5)
F7	0.16383 (14)	0.12649 (15)	0.1440 (2)	0.0607 (7)	
F8	0.14807 (14)	0.19481 (11)	−0.0251 (3)	0.0595 (7)	
F9	0.11541 (12)	0.09611 (11)	−0.0743 (2)	0.0456 (6)	
F10	0.3779 (2)	−0.04902 (12)	−0.0768 (3)	0.0724 (8)	
F11	0.30798 (17)	0.00107 (16)	−0.2707 (3)	0.0828 (10)	
F12	0.43615 (16)	0.01590 (12)	−0.1746 (3)	0.0568 (7)	

### Atomic displacement parameters (Å^2^)

**Table:**

	*U*^11^	*U*^22^	*U*^33^	*U*^12^	*U*^13^	*U*^23^
Sn1	0.02302 (16)	0.02508 (18)	0.02467 (17)	0.000	0.00986 (12)	0.000
C1	0.0201 (14)	0.0296 (17)	0.0228 (15)	0.0012 (13)	0.0078 (12)	−0.0006 (13)
C2	0.0345 (18)	0.0299 (19)	0.0295 (17)	−0.0011 (14)	0.0128 (14)	−0.0008 (14)
C3	0.040 (2)	0.040 (2)	0.0312 (18)	−0.0052 (16)	0.0169 (16)	−0.0034 (16)
C4	0.041 (2)	0.044 (2)	0.0323 (18)	−0.0032 (17)	0.0187 (16)	−0.0084 (16)
C5	0.0286 (17)	0.0303 (18)	0.0311 (17)	−0.0019 (14)	0.0096 (14)	−0.0071 (15)
C6	0.0245 (16)	0.0284 (17)	0.0272 (16)	0.0001 (14)	0.0082 (13)	−0.0012 (14)
C7	0.074 (3)	0.048 (3)	0.047 (2)	−0.003 (2)	0.040 (2)	0.002 (2)
C8	0.054 (2)	0.031 (2)	0.045 (2)	−0.0021 (19)	0.022 (2)	−0.0098 (18)
C9	0.0243 (15)	0.0221 (16)	0.0216 (15)	−0.0004 (12)	0.0086 (12)	0.0005 (12)
C10	0.0243 (15)	0.0240 (16)	0.0212 (15)	0.0004 (13)	0.0079 (12)	0.0012 (13)
C11	0.0257 (15)	0.0233 (16)	0.0217 (15)	0.0023 (13)	0.0087 (12)	0.0038 (13)
C12	0.0270 (16)	0.0260 (17)	0.0214 (15)	−0.0001 (13)	0.0060 (12)	0.0016 (13)
C13	0.0319 (17)	0.0243 (17)	0.0264 (16)	0.0036 (14)	0.0108 (13)	−0.0021 (13)
C14	0.0262 (16)	0.0276 (17)	0.0293 (16)	0.0009 (13)	0.0120 (13)	−0.0002 (14)
C15	0.0275 (17)	0.036 (2)	0.0311 (17)	−0.0016 (15)	0.0099 (14)	−0.0050 (15)
C16	0.035 (2)	0.038 (2)	0.047 (2)	0.0041 (16)	0.0125 (17)	−0.0124 (18)
F1	0.232 (14)	0.034 (4)	0.131 (10)	−0.017 (7)	0.149 (11)	−0.013 (6)
F2	0.033 (3)	0.096 (6)	0.061 (4)	−0.002 (3)	0.028 (3)	0.017 (4)
F3	0.050 (4)	0.204 (12)	0.041 (3)	−0.010 (5)	0.015 (3)	0.054 (5)
F1A	0.058 (4)	0.083 (7)	0.081 (5)	−0.038 (4)	0.023 (4)	0.018 (4)
F2A	0.314 (18)	0.066 (6)	0.175 (13)	−0.029 (10)	0.215 (13)	−0.028 (8)
F3A	0.084 (6)	0.194 (12)	0.080 (7)	−0.029 (6)	0.019 (5)	0.094 (8)
F4	0.127 (8)	0.037 (5)	0.227 (14)	0.020 (5)	0.128 (9)	−0.004 (10)
F5	0.118 (8)	0.044 (4)	0.041 (4)	−0.003 (5)	−0.013 (4)	−0.012 (3)
F6	0.105 (5)	0.029 (4)	0.058 (5)	−0.003 (3)	0.036 (5)	−0.005 (3)
F4A	0.135 (12)	0.044 (8)	0.108 (11)	−0.004 (10)	0.098 (9)	−0.026 (7)
F5A	0.060 (7)	0.030 (5)	0.110 (12)	−0.026 (4)	0.037 (7)	−0.030 (6)
F6A	0.149 (14)	0.045 (8)	0.041 (7)	0.028 (9)	−0.030 (9)	0.012 (6)
F7	0.0406 (13)	0.110 (2)	0.0408 (13)	0.0007 (14)	0.0263 (10)	−0.0102 (14)
F8	0.0309 (12)	0.0405 (14)	0.104 (2)	0.0091 (10)	0.0197 (13)	0.0040 (13)
F9	0.0249 (10)	0.0544 (14)	0.0565 (14)	−0.0069 (10)	0.0125 (9)	−0.0180 (11)
F10	0.107 (2)	0.0271 (13)	0.110 (2)	0.0066 (14)	0.072 (2)	−0.0060 (14)
F11	0.0602 (17)	0.101 (2)	0.0673 (18)	0.0213 (16)	−0.0035 (14)	−0.0584 (17)
F12	0.0640 (16)	0.0546 (16)	0.0696 (16)	−0.0009 (12)	0.0458 (14)	−0.0217 (13)

### Geometric parameters (Å, °)

**Table:**

Sn1—C9	2.146 (3)	C8—F5	1.302 (11)
Sn1—C9^i^	2.146 (3)	C8—F4	1.313 (12)
Sn1—C1	2.150 (3)	C8—F4A	1.314 (16)
Sn1—C1^i^	2.150 (3)	C8—F5A	1.346 (14)
C1—C6	1.391 (5)	C8—F6	1.351 (10)
C1—C2	1.392 (5)	C9—C14	1.393 (4)
C2—C3	1.393 (5)	C9—C10	1.393 (4)
C2—H2A	0.9500	C10—C11	1.393 (4)
C3—C4	1.387 (5)	C10—H10A	0.9500
C3—C7	1.504 (5)	C11—C12	1.386 (4)
C4—C5	1.390 (5)	C11—C15	1.489 (5)
C4—H4A	0.9500	C12—C13	1.389 (4)
C5—C6	1.391 (5)	C12—H12A	0.9500
C5—C8	1.497 (5)	C13—C14	1.388 (5)
C6—H6A	0.9500	C13—C16	1.493 (5)
C7—F1	1.220 (10)	C14—H14A	0.9500
C7—F2A	1.231 (9)	C15—F7	1.323 (4)
C7—F3	1.321 (7)	C15—F9	1.330 (4)
C7—F3A	1.331 (7)	C15—F8	1.352 (4)
C7—F1A	1.351 (10)	C16—F11	1.328 (4)
C7—F2	1.381 (8)	C16—F12	1.328 (4)
C8—F6A	1.239 (14)	C16—F10	1.337 (5)
			
C9—Sn1—C9^i^	109.73 (16)	F6A—C8—F4A	111.1 (12)
C9—Sn1—C1	104.69 (11)	F6A—C8—F5A	109.0 (12)
C9^i^—Sn1—C1	108.35 (11)	F4A—C8—F5A	104.5 (9)
C9—Sn1—C1^i^	108.35 (11)	F5—C8—F6	104.9 (7)
C9^i^—Sn1—C1^i^	104.69 (11)	F4—C8—F6	104.3 (8)
C1—Sn1—C1^i^	120.82 (17)	F6A—C8—C5	111.7 (11)
C6—C1—C2	118.6 (3)	F5—C8—C5	114.3 (8)
C6—C1—Sn1	125.7 (2)	F4—C8—C5	112.2 (9)
C2—C1—Sn1	115.6 (2)	F4A—C8—C5	112.6 (15)
C1—C2—C3	121.0 (3)	F5A—C8—C5	107.6 (9)
C1—C2—H2A	119.5	F6—C8—C5	113.2 (6)
C3—C2—H2A	119.5	C14—C9—C10	118.0 (3)
C4—C3—C2	120.0 (3)	C14—C9—Sn1	121.2 (2)
C4—C3—C7	120.1 (3)	C10—C9—Sn1	120.7 (2)
C2—C3—C7	119.9 (4)	C11—C10—C9	121.1 (3)
C3—C4—C5	119.4 (3)	C11—C10—H10A	119.5
C3—C4—H4A	120.3	C9—C10—H10A	119.5
C5—C4—H4A	120.3	C12—C11—C10	120.5 (3)
C4—C5—C6	120.4 (3)	C12—C11—C15	120.2 (3)
C4—C5—C8	119.5 (3)	C10—C11—C15	119.3 (3)
C6—C5—C8	120.1 (3)	C11—C12—C13	118.8 (3)
C1—C6—C5	120.6 (3)	C11—C12—H12A	120.6
C1—C6—H6A	119.7	C13—C12—H12A	120.6
C5—C6—H6A	119.7	C14—C13—C12	120.8 (3)
F1—C7—F2A	119.8 (9)	C14—C13—C16	120.1 (3)
F1—C7—F3	110.7 (7)	C12—C13—C16	119.0 (3)
F2A—C7—F3A	109.0 (6)	C13—C14—C9	120.9 (3)
F2A—C7—F1A	107.7 (6)	C13—C14—H14A	119.6
F3—C7—F1A	130.4 (9)	C9—C14—H14A	119.6
F3A—C7—F1A	101.6 (6)	F7—C15—F9	106.7 (3)
F1—C7—F2	106.6 (6)	F7—C15—F8	106.3 (3)
F3—C7—F2	100.6 (5)	F9—C15—F8	105.9 (3)
F3A—C7—F2	133.7 (7)	F7—C15—C11	112.3 (3)
F1—C7—C3	116.1 (6)	F9—C15—C11	113.4 (3)
F2A—C7—C3	117.1 (7)	F8—C15—C11	111.6 (3)
F3—C7—C3	111.8 (6)	F11—C16—F12	105.9 (3)
F3A—C7—C3	109.2 (6)	F11—C16—F10	108.0 (3)
F1A—C7—C3	111.2 (7)	F12—C16—F10	104.7 (3)
F2—C7—C3	109.6 (6)	F11—C16—C13	112.5 (3)
F6A—C8—F5	116.9 (15)	F12—C16—C13	113.5 (3)
F5—C8—F4	107.2 (7)	F10—C16—C13	111.7 (3)

Symmetry codes: (i) −*x*+1, *y*, −*z*+1/2.
